# A novel approach for the management of infrabony periodontal defects using autologous dentin and L-PRF: a clinical case series report

**DOI:** 10.3389/froh.2026.1755090

**Published:** 2026-04-08

**Authors:** A. Anwandter-Beckhaus, M. J. Orlandini, D. Prieto, N. Pinto, A. Celis, A. Sanz, C. Martínez-Cardozo

**Affiliations:** 1 Department of Pathology and Conservative Dentistry, Faculty of Dentistry, Universidad de los Andes, Santiago, Chile; 2 Postgraduate Program in Periodontics, Faculty of Dentistry, Universidad de los Andes, Santiago, Chile; 3 Department of Diagnosis and Surgical Sciences, Faculty of Dentistry, Universidad de los Andes, Santiago, Chile; 4 Department of Growth, Development and Public Health, Faculty of Dentistry, Universidad de los Andes, Santiago, Chile; 5 Department of Biomedical Sciences, Ethics, Research and Education, Faculty of Dentistry and Center for Biomedical Research and Innovation (CIIB), Universidad de Los Andes, Santiago, Chile

**Keywords:** autologous, dentin, periodontal diseases, platelet-rich fibrin, treatment

## Abstract

**Background:**

Autologous dentin has emerged as a biologically active alternative to xenogeneic grafts, yet its clinical application in periodontal healing remains limited. Leukocyte- and platelet-rich fibrin (L-PRF) provides a scaffold that releases biomolecules to enhance wound healing. This case series evaluates the clinical and radiographic outcomes of a fully autologous L-PRF dentin block for the treatment of infrabony periodontal defects.

**Methods:**

Six patients presenting with twenty infrabony defects were treated with an L-PRF dentin block graft composed of particulate autologous dentin combined with L-PRF membranes and liquid fibrinogen. At baseline and after 6 months, Clinical attachment level (CAL), probing depth (PD), and bleeding on probing (BOP) were evaluated clinically, and defect dimensions were assessed by Cone-beam computed tomography (CBCT). Pain was evaluated using a visual analog scale at 0, 7, 14, and 21 days.

**Results:**

Significant improvements were observed in all clinical parameters at 6 months: CAL decreased from 5.7 mm (95% CI: 5.1–6.2) to 1.6 mm (95% CI: 0.8–2.4; *p* < 0.0001), PD decreased from 7.2 mm (95% CI: 6.2–8.2) to 3.7 mm (95% CI: 3.0–4.3; *p* < 0.0001), and BOP from 80% to 10% (*p* < 0.0001). CBCT analyses demonstrated significant reductions in defect depth (*p* < 0.03) and mesiodistal and buccopalatal dimensions (*p* < 0.0001). Patients reported low postoperative pain from day 7 onward.

**Conclusion:**

The L-PRF dentin block represents a promising, fully autologous, biologically based approach for the treatment of infrabony periodontal defects, associated with favorable preliminary clinical and radiographic outcomes with minimal postoperative discomfort. These findings suggest that this approach may represent a feasible and biologically compatible alternative to conventional graft materials. However, randomized clinical trials are needed to confirm these preliminary findings.

## 1 Introduction

Periodontitis is a complex, multicausal inflammatory disease that affects a large portion of the global population and leads to the progressive loss of periodontal tissues. This condition can lead to gingival recession, increased tooth sensitivity, tooth mobility, bone destruction, and ultimately, tooth loss (1). Particularly, the loss of clinical attachment and bone resorption can present in a horizontal or vertical pattern, defined as vertical bony or infrabony defects characterized by one-wall, two-wall, three-wall, or combined defects (2). Infrabony periodontal defect treatment aimed to achieve proper periodontal regeneration, including the formation of new periodontal ligament, cementum, bone, and connective tissue attachment to support long-term periodontal stability (3). Over the past four decades, a wide range of biomaterials, biologic agents, and membranes have been employed in efforts to regenerate periodontal tissues affected by periodontal disease that generate these infrabony defects (4). The use of biological factors has gained interest due to their beneficial role in defect healing, as demonstrated by clinical and radiographic outcomes (5). Biologic agents, such as recombinant platelet-derived growth factor (rhPDGF-BB), enamel-derived proteins (EMD), and autologous platelet-derived concentrates such as Leukocyte and platelet-rich fibrin (L-PRF), as well as hyaluronic acid (HA), which has recently emerged as a bioactive adjunct particularly when combined with xenografts or barrier membranes (6) and have proven effective in managing infrabony periodontal defects, improving the clinical periodontal parameters, such as clinical attachment level (CAL) gain and probing depth (PD) reduction, as well as radiographic parameters, including defect fill, especially when combined with graft biomaterials, including bovine or human bone (7, 8). Hyaluronic acid has been shown to modulate inflammation, enhance angiogenesis, and promote cell migration and early wound stability, with clinical studies reporting improved outcomes in the treatment of intrabony defects when used alone or in combination with bone substitutes.

Reported clinical improvements in regenerative approaches for intrabony defects generally range between approximately 1.5–5 mm of CAL gain and 1.5–6 mm of PD reduction, depending on defect morphology, surgical technique, and the use of adjunctive biologically active materials (9).

L-PRF is a second generation of autologous platelet concentrates. Its beneficial effects on tissue healing have been attributed to its temporary fibrin matrix and the sustained release of growth factors and other bioactive molecules, including PDGF isoforms (PDGF-AA/AB/BB), transforming growth factor beta (TGF-β), vascular endothelial growth factor (VEGF), and epidermal growth factor (EGF) (10). These mediators promote angiogenesis and support the migration, proliferation, and differentiation of mesenchymal progenitor cells, fibroblasts, periodontal ligament cells, and osteoblasts, as evidenced in previous *in vitro* studies (11–15). From a periodontal perspective, the coordinated formation of new cementum and a functional periodontal ligament attachment depends on tightly regulated cell–matrix interactions and growth factor signaling. Cementoblast precursor cells are influenced by multiple growth factors, and the establishment of a new fibrous attachment is driven by extracellular matrix cues, integrins, and growth factor pathways that could be released from L-PRF (10).

A recent systematic review that included at least 20 randomized clinical trials indicates that adding L-PRF to the surgical treatment of intrabony defects provides additional clinical benefits. On average, a PD reduction of about 1.3 mm, a CAL gain of approximately 1.2 mm, and a radiographic bone gain of around 1.5 mm were observed when L-PRF was used following open flap debridement (8). When L-PRF was combined with bone grafts, outcomes were generally better than with grafts alone. The additional improvements were more modest, averaging about 0.6 mm in PPD reduction, CAL gain, and radiographic bone fill. Although L-PRF does not maintain space as effectively as a bone graft, most studies have shown no significant difference between PRF alone and bone grafts alone, suggesting that both provide benefits through different biological mechanisms (8). Overall, the evidence suggests that combination therapies (bone graft + L-PRF) tend to produce superior clinical outcomes compared with single-modality approaches, which aligns with recent evidence-based reviews supporting the use of biologically enhanced regenerative strategies for intrabony defect treatment (5, 8).

Although most bone substitute biomaterials used for periodontal treatment fill the defect and primarily exhibit osteoconductive and low osteoinductive properties, previous studies indicates that, under favorable defect configurations and biologically supportive conditions, some biomaterials may contribute to true periodontal regeneration, including new cementum, functionally oriented periodontal ligament fibers, and alveolar bone formation, evidenced by histological analyses (9). Similarly, biomaterials containing bovine bone often exhibit limited biodegradability and prolonged persistence at the grafted site, lasting up to 11 years, and in some cases, provoking chronic inflammatory responses characterized by multinucleated giant cells and cysts (16–18).

In recent years, autologous dentin particulate has gained attention as a promising alternative to xenogeneic graft materials. Derived from extracted teeth, dentin shares several biochemical and structural similarities with bone, making it a biologically favorable option for regenerative procedures. One of the most significant advantages of dentin particulate is its osteoconductive and osteoinductive potential. Dentin contains high levels of type I collagen, hydroxyapatite, and a variety of growth factors, including bone morphogenetic proteins (BMP-2, BMP-7), transforming growth factor beta (TGF-β), and insulin-like growth factor (IGF), which are released in a controlled manner during its resorption. These factors contribute to the active stimulation of osteoblast/cementoblast differentiation and the enhanced recruitment of progenitor cells, leading to true osteoinduction, which differs from the osteoconductive properties of biomaterials (19). Despite the growing body of evidence on biologically active adjuncts and alternative grafting materials, clinical data on the combined use of autologous dentin and platelet concentrates in periodontal intrabony defects remain scarce. While previous research has demonstrated the regenerative potential of autologous dentin combined with L-PRF primarily in the context of alveolar ridge preservation following tooth extraction, the present study is, to our knowledge, among the first to report the clinical application of this biologically active combination in the treatment of periodontal infrabony defects. This case series documents the successful use of ground autologous dentin and L-PRF membranes in a L-PRF dentin block for the treatment of twenty infrabony periodontal defects in six patients, demonstrating consistent improvements in clinical attachment levels, reduction in probing depth, and radiographic bone fill after 6 months. Our findings expand the potential indications of dentin L-PRF matrices beyond socket preservation and highlight their promising role in periodontal healing, where tissue architecture is more complex and biologically demanding.

## 2 Case series report

### 2.1 Participants enrollment

Patients were recruited via convenience sampling between 2019 and 2021 from the Universidad de los Andes Health Center (CESA), where they were treated as part of the postgraduate periodontics program. The inclusion criteria were as follows: patients over 18 years of age, of any gender, with no medical or psychiatric conditions that would contraindicate surgical treatment (i.e., ASA class III or above), who required extraction of at least one tooth (non-endodontically treated), and presented with at least one periodontal infrabony defect on a different tooth than the one to be extracted. Eligible infrabony defects included two- or three-wall defects of ≥3 mm in depth and an angle ≤30°, as assessed by periapical radiographs. Anterior and posterior teeth without furcation involvement were included. Exclusion criteria included pregnancy, systemic conditions that may impair bone healing (e.g., osteoporosis under bisphosphonate treatment, uncontrolled diabetes with HbA1c > 7%), psychiatric disorders, poor plaque control (plaque index >30%), and heavy smoking (>10 cigarettes/day). The study protocol was approved by the Ethics Committee of Universidad de los Andes, and all participants provided written informed consent voluntarily.

### 2.2 Clinical and radiographic assessment

For each participant, a comprehensive clinical chart was completed, documenting gender, age, medical history, social habits, and a full-mouth periodontal chart. Periapical radiographs and CBCT scans of the surgical site were taken as part of a comprehensive treatment plan. Intraoral and extraoral photographs were also recorded. Clinical attachment levels (CAL), bleeding on probing (BOP), and probe depth (PD), were taken at baseline and after six months of surgical treatment. A calibrated examiner performed all clinical measurements with an intra-examiner kappa value of 0.89. Cone-beam computed tomography (CBCT) scans were obtained at baseline and 6 months after surgical therapy to assess changes. Radiographic analysis included (defect depth, mesiodistal, and vestibulo-palatal distance in mm) and was performed by a calibrated examiner with a kappa value of 0.96.

All participants received non-surgical periodontal therapy, and those with adequate periodontal health (PI < 20% and GI < 1) proceeded to surgery. Teeth with an indication for extraction were extracted before periodontal surgical procedures.

### 2.3 Preparation of L-PRF dentin block for surgical procedure

**Figure 1:**
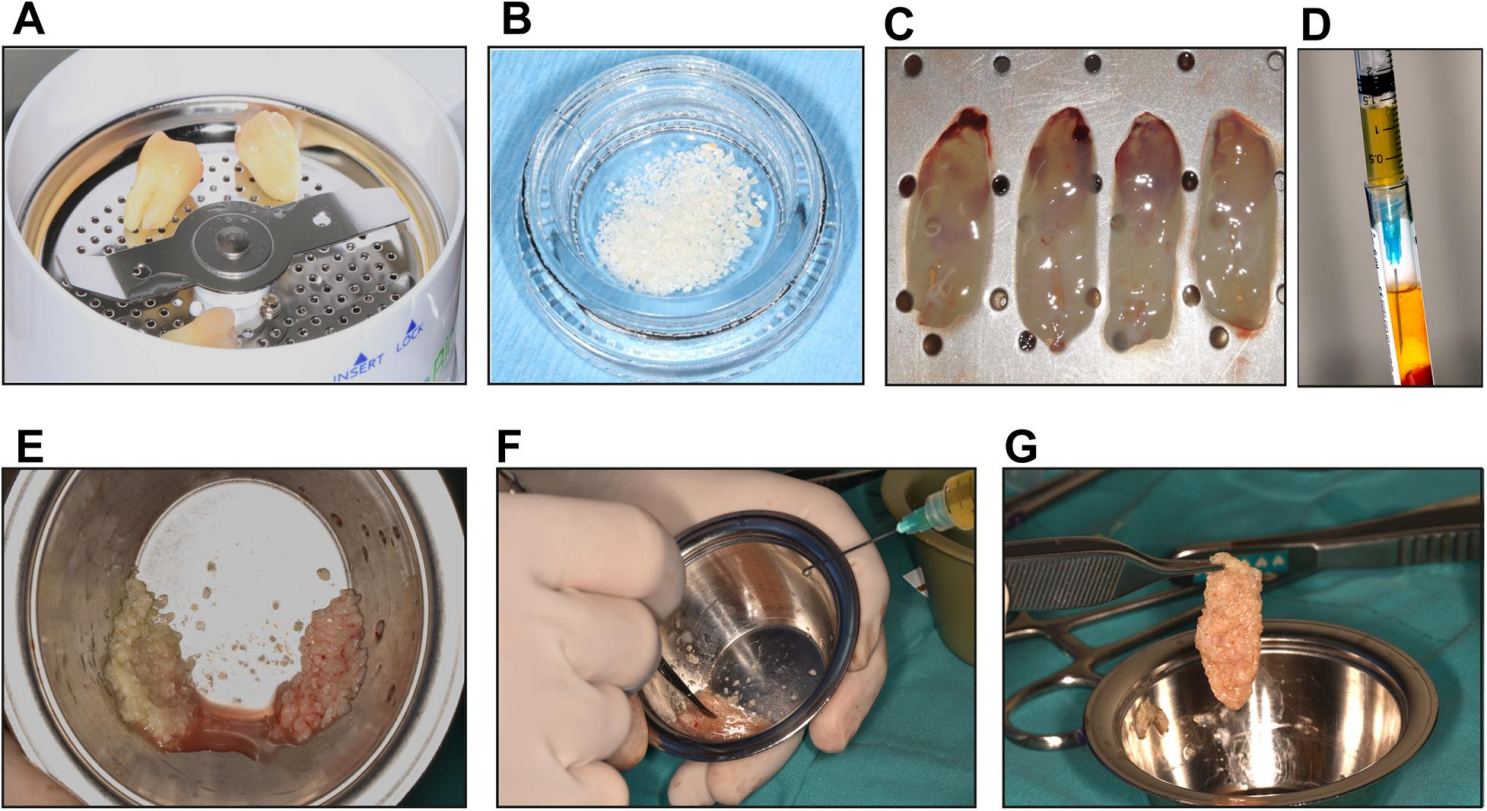
Preparation of the L-PRF dentin block. Representative images of the process for L-PRF and particulate dentin obtaining. **(A)** Extracted teeth were cleaned and processed by the *Smart Dentin Grinder™* (Kometa Bio®). **(B)** Particulated dentin material immediately after processing. **(C)** L-PRF membranes after 5 min of compression using the *Xpression® box*. **(D)** Collection of liquid fibrinogen using a tuberculin syringe. **(E)** L-PRF membranes were cut into small fragments and mixed with the particulate dentin. **(F)** L-PRF and dentin were mixed with liquid fibrinogen to get the L-PRF dentin block. **(G)** Fully prepared dentin block.

The extraction of the non-restorable tooth was performed approximately two weeks before the periodontal defect treatment. Any soft-tissue remnants, restorations, and foreign materials were removed using turbine diamond burs and tungsten carbide burs. The tooth was subsequently cleaned and immersed in 70% ethanol until further processing, as previously reported (20). On the day of the periodontal defect surgery, the tooth was mechanically processed using the Smart Dentin Grinder™ system (Figure 1A) to obtain particulate dentin (300–1200 µm). The particles were decontaminated using Dentin Cleanser for 5 min, then demineralized and neutralized with phosphate-buffered saline (PBS) for 2 min, according to the manufacturer's protocol (Figure 1B). For preparation of the L-PRF dentin block to treat the infrabony defect, with 4 to 6 L-PRF membranes and 2 tubes of liquid fibrinogen were prepared from each patient. L-PRF membranes were obtained by centrifuging 10 mL of venous blood in red-top tubes (without anticoagulant) for 12 min at 2700 rpm using an IntraSpin centrifuge (IntraLock, Alabama, USA). L-PRF clots were taken from the middle part of the tubes and then gently compressed for 5 min using an Xpression® box (IntraSpin, Biohorizons, Alabama, USA) to get L-PRF membranes (Figure 1C). Liquid fibrinogen was obtained by centrifuging 9 mL of venous blood in white-top tubes (without anticoagulants) for 3 min at 2700 rpm (Figure 1D). After that, two L-PRF membranes were finely chopped with surgical scissors and then mixed with 0.5 g of particulate dentin and liquid fibrinogen. The mixture was gently stirred for approximately 60 s until a cohesive graft mass was achieved (Figures 1E, F). The resulting dentin L-PRF block graft material exhibited a moldable, cohesive consistency, allowing easy adaptation to the geometry of the periodontal defects (Figure 1G).

### 2.4 L-PRF dentin block surgical procedure

**Figure 2:**
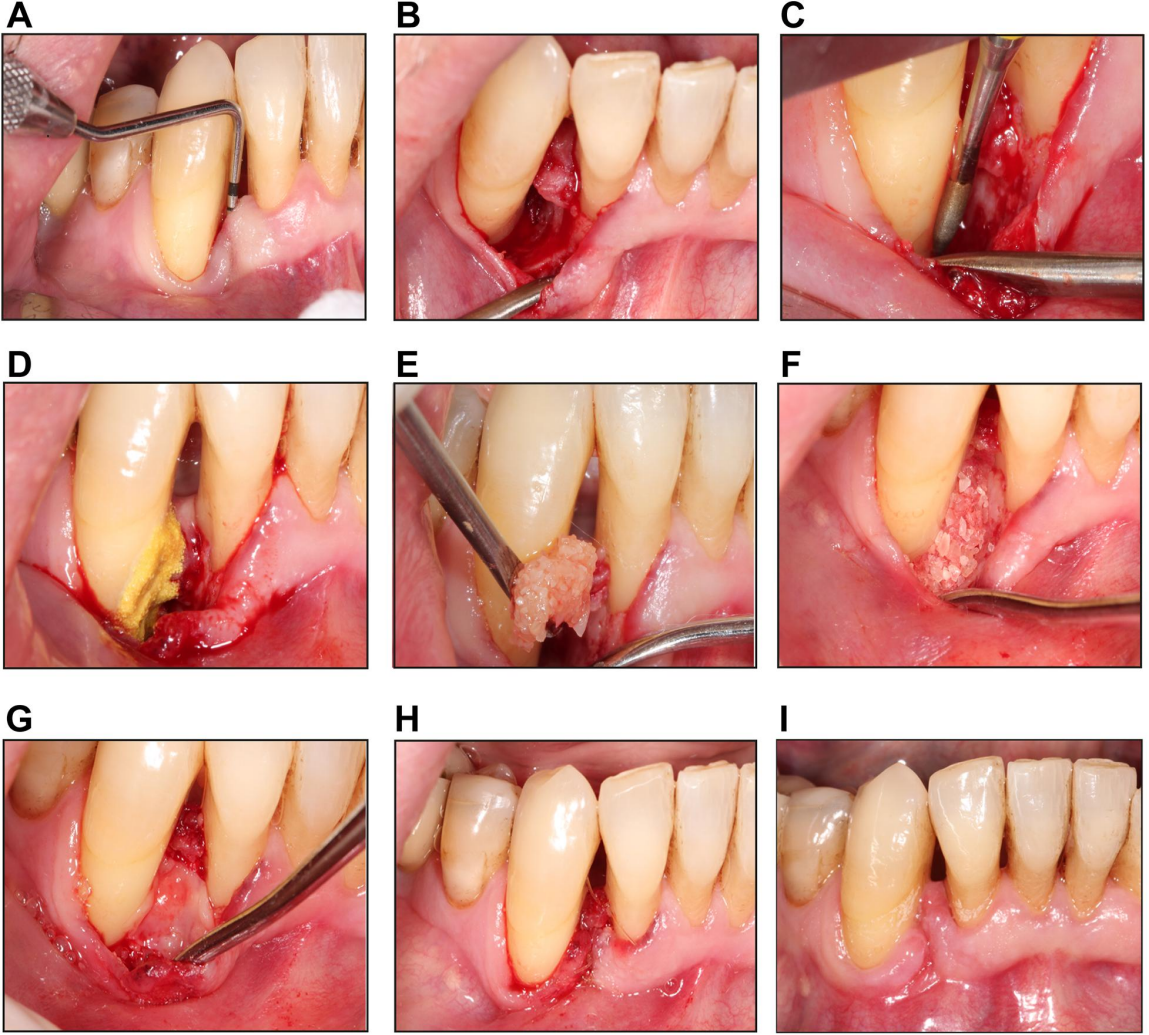
Surgical technique for treating infrabony periodontal defects with L-PRF and autologous dentin. Representative images of **(A)** initial probing depth measured with a North Carolina periodontal probe (Hu-Friedy®), **(B)** buccal access flap using the M-MIST technique showing the infrabony defect. **(C)** Final polishing of the root surface with fine-grit burs (PerioKit) under saline irrigation. **(D)** Application of tetracycline hydrochloride diluted in saline for 2 min. **(E,F)** The defect was filled with a grafting material composed of L-PRF and autologous dentin. **(G)** L-PRF membrane covering the dentin graft before suturing. **(H)** Internal modified vertical mattress suture using Monosyn 5-0. **(I)** Postsurgical control after seven days of the surgery.

All surgical procedures were performed by the same operator (AA). Under local anesthesia, a full thickness intracrevicular flap was raised at the involved tooth or teeth. When the defect was limited to the buccal aspect and accessible only from that side, a modified minimally invasive surgical technique (M-MIST) was employed (Figures 2A, B). In contrast, when the defect involved three or four walls, a wider flap was designed to include adjacent teeth, with consideration given to both the buccal and palatal/lingual aspects. Granulation tissue was thoroughly removed, and root planning was performed under direct vision using rigid Gracey curettes (Hu-Friedy®, Chicago, USA). Final root surface polishing was completed using fine-grit burs (PerioKit, Jota, Rüthi, Switzerland) at a maximum speed of 2,000 RPM under copious saline irrigation (Figure 2C). The root surface was then conditioned with a paste of tetracycline hydrochloride (250 mg) mixed with sterile saline, applied for 2 min, and rinsed with sterile saline (Figure 2D). The defect was subsequently filled with the Dentin L-PRF Block graft material (Figures 2E, F). This was covered with an L-PRF membrane, and the flap was repositioned and sutured using Monosyn 5–0 sutures (B. Braun, Hessen, Germany) in a modified internal vertical mattress technique (Figures 2G, H).

All patients were instructed to avoid brushing the surgical area and to rinse twice daily with 0.12% chlorhexidine mouthwash (PerioAid, Dentaid, Cerdanyola, Barcelona) for 2 weeks. Analgesic management consisted of acetaminophen 1 g, and ibuprofen 400 mg, both every 8 h for 5 days.

### 2.5 Follow-up assessments and data analysis

Postoperative check-ups were scheduled at 7, 14, and 21 days, and a visual analog scale (VAS) was used to evaluate pain associated with the procedures. Sutures were removed at 14 days. A six-month follow-up, including the assessment of periodontal clinical parameters and CBCT imaging, was done. All data were entered into an Excel® sheet (Microsoft®), and statistical analysis was performed using a mixed-effects model to account for the correlated structure of repeated observations within individuals and within defects. Clinical and CBCT measures were analyzed at the defect level across follow-up (baseline vs. 6 months), with a random intercept for participant and repeated measures specified at the defect level nested within participant. Changes between baseline and 6 months were analysed using linear mixed-effects models, with a random intercept for participant and repeated-measures structure specified at the defect level nested within participant, using an unstructured covariance matrix. Estimates were reported as least-squares means, with their differences and 95% confidence intervals, and two-sided *p*-values. Degrees of freedom and standard errors for fixed-effect inference were estimated using the Kenward–Roger method (*α* = 0.05). All models met convergence criteria. Statistical analyses were performed using SAS software version 9.4 (SAS Institute Inc., Cary, NC, USA). For graphical illustration of the distribution and direction of changes in clinical parameters, exploratory paired nonparametric comparisons were used solely for visualization (Wilcoxon matched-pairs signed rank test). These analyses were not considered confirmatory and were intended solely to support the interpretation of the mixed-effects model results. For VAS scale data, non-parametric analyses were performed using the Kruskal–Wallis test (Supplementary Figure S1). Wilcoxon and Kruskal–Wallis analyses were conducted using GraphPad Prism software (version 10).

## 3 Clinical and imaging findings

Six patients were included, comprising three females and three males. The demographic characteristics of the patients are summarized in Table 1. Patient ages ranged from 33 to 68 years, with a mean age of 54.8 years. None of the patients reported any systemic diseases or ongoing medication use. Two patients reported tobacco use, both consuming approximately one cigarette per day. No patients reported recreational drug use. A total of 20 periodontal defects were included. Three patients presented with a single defect, whereas the remaining patients exhibited 3, 5, and 9 defects, respectively (Supplementary Table S1). All surgical procedures were carried out as planned, with no intraoperative complications. The average pain reported by participants, measured using the visual analog scale, was 3.66 (SD ± 1.506) on the first day. Participants reported significant reductions in or no pain at follow-up evaluations on days 7, 14, and 21 (Supplementary Figure S1).

**Table 1:** Clinical and imaging defect analyses.

Patients	Gender	Age mean	Smoking	Total Defects
6	3 F 3 M	54.8 +/- 13.2	33%	*n* = 20
**Defect Analysis**				
Clinical	Baseline mean (CI 95%) [SE]	6 months mean (CI 95%) [SE]	Difference (CI 95%) [SE]	*P* value
CAL (mm)	5.7 (5.1; 6.2) [0.3]	1.6 (0.8; 2.4) [0.4]	4.1 (2.9; 5.2) [0.5]	<0.0001
PD (mm)	7.2 (6.2; 8.2) [0.5]	3.7 (3.0; 4.3) [0.2]	3.5 (2.6; 4.4) [0.4]	<0.0001
BOP (%)	80 (61; 92) [0.1]	10 (0.4; 24) [0.1]	70 (43; 97) [0.1]	<0.0001
Clinical Change	PPD Reduction (mm) Mean (CI 95%) [SE]	CAL Gain (mm) Mean (CI 95%) [SE]		
	3.5 (2.6; 4.4) [0.4]	4.1 (2.9; 5.2) [0.5]		
CBCT	Baseline mean (CI 95%) [SE]	6 months mean (CI 95%) [SE]	Difference (CI 95%) [SE]	*P* value
Deep Length (mm)	4.2 (2.0; 6.4) [0.8]	3.0 (0.6; 5.4) [0.8]	1.2 (0.6; 1.8) [0.3]	0.003
MD length (mm)	2.0 (1.1; 2.9) [0.3]	1.0 (0.1; 1.9) [0.3]	1.0 (0.6; 1.4) [0.2]	<0.0001
BP length (mm)	5.5 (4.2; 6.7) [0.5]	2.5 (1.2; 3.7) [0.5]	3.0 (2.3; 3.7) [0.3]	<0.0001

Six patients (three women and three men) with 20 infrabony defects were included. Defects were analyzed using Clinical Parameters: clinical attachment level (CAL), bleeding on probing (BOP), probing depth (PD) and CBCT measurements: Mesial-Distal (MD), Bucco-Palatal (BP). A mixed-effects model was fitted to account for the correlated structure of repeated observations within individuals and within defects. Estimates are reported as least-squares means, their between-time differences, 95% confidence intervals, and two-sided *p*-values. Denominator degrees of freedom were estimated using the Kenward–Roger method (*α* = 0.05), yielding 19 degrees of freedom for all models.

**Figure 3:**
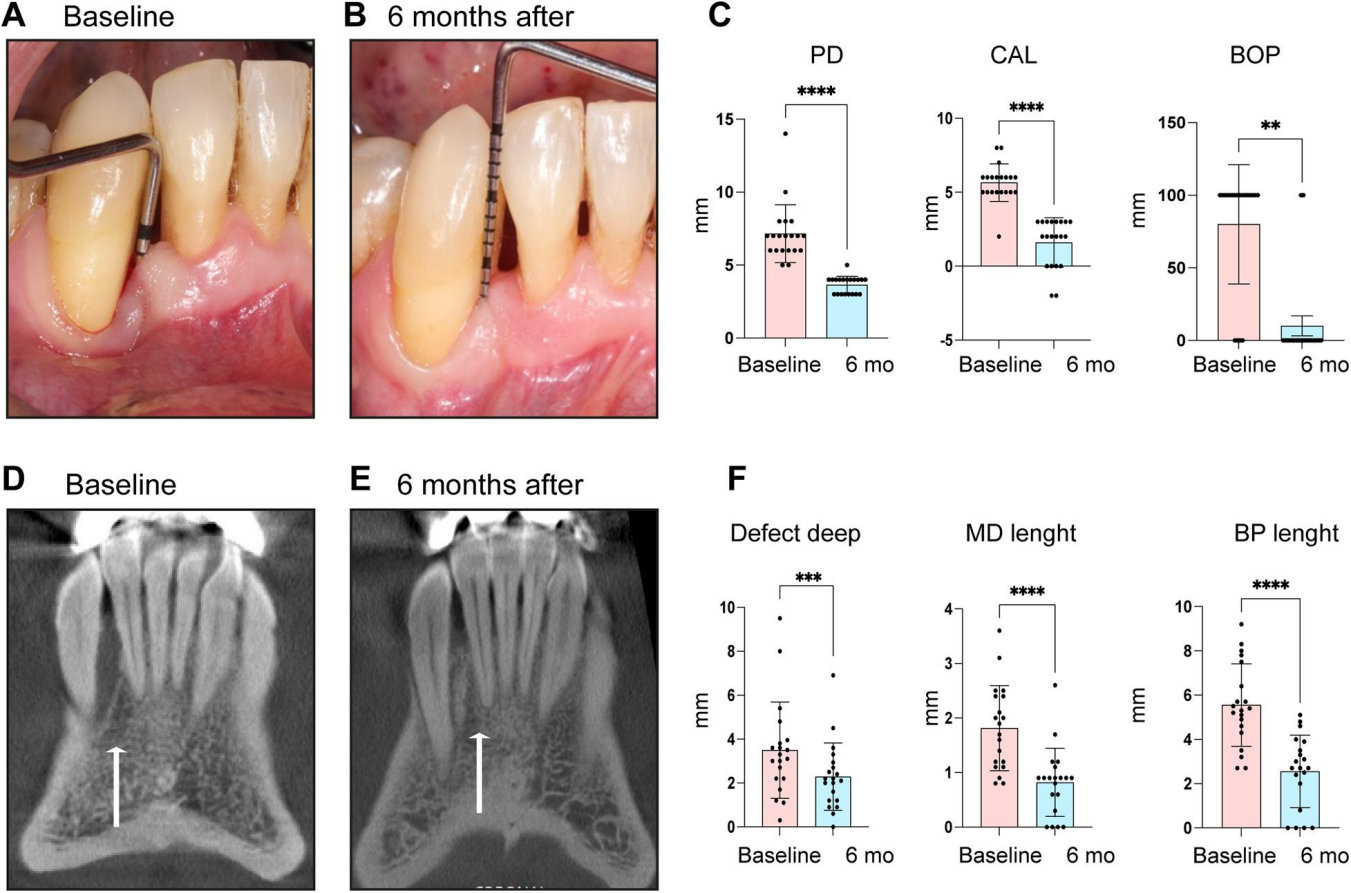
L-PRF dentin block treatment improves clinical and CBCT imaging parameters following treatment of infrabony defects. Representative images of **(A)** periodontal infrabony defect at baseline, and **(B)** six-month follow-up. **(C)** Quantification of the periodontal clinical parameters (PD, CAL, BOP) at baseline and after six months. Representative images of the coronal CBCT views of the infrabony defect at **(D)** baseline and **(E)** six-month follow-up. **(F)** Quantification of imaging measurements at baseline and six-month follow-up. PD, probing depth; CAL,  clinical attachment level; BOP, bleeding on probing; MD,  mesio distal; BP, buccal palate. Asterisks indicate results of exploratory paired nonparametric comparisons performed using the Wilcoxon matched pairs signed-rank test and are shown for visualization purposes only.

Significant statistical differences were found between baseline and 6 months after L-PRF dentin block surgical treatment in all clinical and imaging parameters at the 20 evaluated sites (Table 1 and Figure 3). The mean of Clinical attachment levels (CAL) was 5.7 mm (95% CI:5.1–6.2) at baseline and 1.6 mm (95% CI: 0.8–2.4; *p* < 0.0001) at the six-month follow-up. 80% of sites demonstrated bleeding on probing (BOP) at baseline, compared with 10% after 6 months (*p* < 0.0001 Table 1 and Figures 3A–C). Similarly, probing depth (PD) was reduced from 7.2 mm (95% CI: 6.2–8.2) at baseline to 3.7 mm (95% CI:3.0–4.3; *p* < 0.0001) after six months (Table 1 and Figures 3A–C). The mean of PPD reduction for all the defects was 3.5 mm (95% CI:2.6–4.4), and the mean of CAL gain was 4.1 mm (95% CI:2.9–5.2) (Table 1). Regarding PPD reduction, 12 out of 20 defects showed a reduction of 2–3 mm, while 4 defects demonstrated a reduction of 4–5 mm, and 2 defects exhibited a reduction of greater than 5 mm. Only 2 defects showed a limited PPD reduction of 0–1 mm. Similarly, CAL gain was predominantly observed in the moderate-to-high response categories. Nine defects achieved a CAL gain of 2–3 mm, whereas 6 defects showed a gain of 4–5 mm, and 4 defects demonstrated a gain of greater than 5 mm. Only one defect presented a minimal CAL gain of 0–1 mm (Supplementary Table S2).

All imaging parameters evaluated in the CBCT showed a significant improvement after six months of surgical treatment as follows: the mean of baseline deep length (from the base of the defect to the alveolar crest) averaged 4.2 mm (95% CI: 2.0–6.4), compared to 3 mm (95% CI:0.6–5.4; *p* = 0.03) (Table 1 and Figures 3D–F). The mesio-distal (MD) width at baseline was 2 mm (95% CI: 1.1–2.9) compared to 1 mm (95% CI: 0.1–1.9; *p* < 0.0001) (Table 1 and Figures 3D–F) and a bucco-palatal (B-P) width of 5.5 mm (95% CI: 4.2–6.7) at baseline compared to 2.5 mm (95% CI: 1.2–3.7; *p* < 0.0001) after treatment (Table 1 and Figures 3D–F).

## 4 Discussion

In this case report series, twenty infrabony defects from six patients were treated with an autologous L-PRF dentin block, associated with statistically and clinically significant improvements in clinical and radiographic outcomes at six months compared to baseline. Mean clinical attachment level (CAL) gain reached 4.1 mm, while mean probing depth (PPD) reduction was 3.5 mm, accompanied by a marked reduction in bleeding on probing (BOP). Additionally, three-dimensional radiographic analysis using CBCT demonstrated significant reductions in defect depth and width**,** supporting true defect resolution rather than soft tissue adaptation alone. These findings suggest that this fully autologous combination may serve as a safe and biologically active approach for periodontal healing.

The observed positive outcomes may be attributed to the synergistic properties of both components. L-PRF provides a fibrin scaffold enriched with platelets and leukocytes, releasing growth factors that promote cell biological activities, including migration, proliferation, differentiation, and angiogenesis. Multiple systematic reviews and meta-analyses have confirmed that L-PRF enhances clinical attachment gain and bone fill compared with open-flap debridement alone (2, 7, 8). According to the American Academy of Periodontology's best evidence consensus statement on the use of biologics in clinical practice, PRF is one of the best therapies in combination with xenogeneic bone graft to maintain the stability of the gingival margin following regenerative treatment of periodontal infrabony defects (5). However, autologous dentin–based therapies were excluded from this consensus due to limited clinical evidence of their promising biological potential.

Autologous dentin is chemically and structurally similar to bone, containing type I collagen and osteoinductive proteins such as bone morphogenetic proteins. Recent studies have provided strong support for its regenerative capability in alveolar ridge preservation (8, 21, 22). Nevertheless, the application of autologous dentin in the treatment of periodontal infrabony defects remains insufficiently explored. Currently, the clinical application of dentin grafts is primarily limited to dental and maxillofacial surgery, representing a significant translational step toward repurposing extracted teeth as biomaterials for localized bone reconstruction (22, 23). The present case series, therefore, represents a translational extension of this biomaterial into periodontal regenerative therapy, highlighting the novelty of combining autologous dentin with L-PRF in infrabony defects.

Minimally invasive periodontal surgical (MIPS) approaches, including M-MIST, have been associated with favorable clinical outcomes in the treatment of infrabony defects, particularly with respect to CAL gain and PPD reduction, with minimal gingival recession. A systematic review and meta-analysis by Clementini et al. reported pooled mean CAL gains of approximately 3.7 mm and mean PPD reductions of around 4.2 mm following minimally invasive periodontal surgery, with follow-up periods ranging from 6 to 12 months (24). However, the available evidence is derived from heterogeneous surgical protocols that frequently combine xenogeneic grafts, enamel matrix derivatives, membranes, or recombinant biologics, and the paucity of controlled studies comparing minimally invasive surgery alone with adjunctive biologic strategies limits the ability to isolate the specific contribution of surgical access vs. biological augmentation (24).

In this context, the present findings suggest that although wound stability and primary closure are key determinants of periodontal healing, achieving a CAL gain of 4.1 mm at 6 months with a fully autologous biologic combination indicates an enhanced, potentially accelerated healing response. Importantly, this magnitude of early attachment gain is comparable to outcomes reported at longer follow-up periods in randomized controlled trials employing M-MIST alone or in combination with xenogeneic grafts (25), underscoring the potential biological contribution of the L-PRF dentin block. However, the combination of L-PRF and autologous dentin for the infrabony defects has been poorly studied, highlighting the novelty of this case series.

Additionally, a clear gap in the literature emerges regarding patient-reported outcome measurements (PROMs) including satisfaction and quality of life in periodontal regenerative therapy. In the present series, pain was the only PROM evaluated over a 21-day postoperative period, showing relatively low pain levels only on the day of the intervention, which were completely absent in the postoperative follow-ups at days 7, 14, and 21. These findings are consistent with previous reports highlighting reduced postoperative morbidity associated with the use of L-PRF to stimulate healing, further supporting the clinical feasibility of the proposed treatment strategy (26).

Combining autologous dentin with L-PRF to create the L-PRF dentin block may therefore benefit two biologically autologous components: one providing a mineralized scaffold, and the other releasing bioactive signals that enhance cellular recruitment and tissue maturation. This dual mechanism may help explain the favorable outcomes observed in the present case series. A limitation of the present case series is the 6-month follow-up period, which does not allow definitive conclusions regarding the long-term stability of the observed regenerative outcomes. The present findings should be interpreted as reflecting an early regenerative response, providing preliminary evidence of the biological and clinical potential of the proposed autologous approach. Future randomized controlled trials with larger cohorts, longer follow-up periods, and histological validation are still needed to confirm the long-term stability and quality of the healing tissues to demonstrate clinical and radiographic findings compatible with regeneration.

Within the limitations of this case series, the autologous L-PRF dentin block appeared to be clinically feasible, biologically compatible, and associated with favorable preliminary clinical and radiographic outcomes in the treatment of periodontal infrabony defects. However, given the limited sample size, short follow-up, and lack of a control group, these findings should be interpreted as exploratory and hypothesis-generating rather than as evidence of clinical effectiveness. Further high-quality evidence is needed to confirm its long-term predictability and cost-effectiveness.

## Data Availability

The original contributions presented in the study are included in the article/Supplementary Material, further inquiries can be directed to the corresponding authors.
