# Supplementary material for: A novel approach for the management of infrabony periodontal defects using autologous dentin and L-PRF: a clinical case series report
Source: Front Oral Health. 2026 Apr 8;7:1755090. doi: 10.3389/froh.2026.1755090 (PMC13099840; doi:10.3389/froh.2026.1755090)
Supplement: Supplementary file 1 [file Table1.docx]

| Patient | Gender | Age | Smoking | Number of defects | Wall number  baseline | Wall number  after treatment |
| --- | --- | --- | --- | --- | --- | --- |
| 1 | F | 57 | No | 3 | 2, 3, 3 | 3, 3, 3 |
| 2 | F | 55 | No | 5 | 3, 2, 2, 2, 2 | 0, 3, 3, 3, 3 |
| 3 | F | 33 | No | 9 | 3, 1, 3, 2, 1,  1, 2, 2, 3 | 3, 1, 3, 0, 3,  2, 0, 3, 3 |
| 4 | M | 68 | Yes | 1 | 2 | 1 |
| 5 | M | 48 | Yes | 1 | 2 | 1 |
| 6 | M | 68 | No | 1 | 2 | 0 |

**Supplementary Table 1. Baseline demographic characteristics and number of periodontal defects per patient.** This table summarizes the demographic variables (gender and age), smoking status, the total number of infrabony defects, and the wall number at baseline and at six months post-treatment, in each patient included in the case series.

| **PPD reduction** | **0-1 mm** | **2-3 mm** | **4-5 mm** | **above 5mm** |
| --- | --- | --- | --- | --- |
| Total defects | 2 | 12 | 4 | 2 |
|  |  |  |  |  |
| **CAL Gain** | **0-1 mm** | **2-3 mm** | **4-5 mm** | **above 5mm** |
| Total defects | 1 | 9 | 6 | 4 |

**Supplementary Table 2. Clinical parameters change in Infrabony defects.** Defects were classified between 0-1mm, 2-3mm, 4-5mm, or above 5mm according to PPD reduction or CAL gain.
