# Supplementary figures and images for: A novel approach for the management of infrabony periodontal defects using autologous dentin and L-PRF: a clinical case series report
Source: Front Oral Health. 2026 Apr 8;7:1755090. doi: 10.3389/froh.2026.1755090 (PMC13099840; doi:10.3389/froh.2026.1755090)

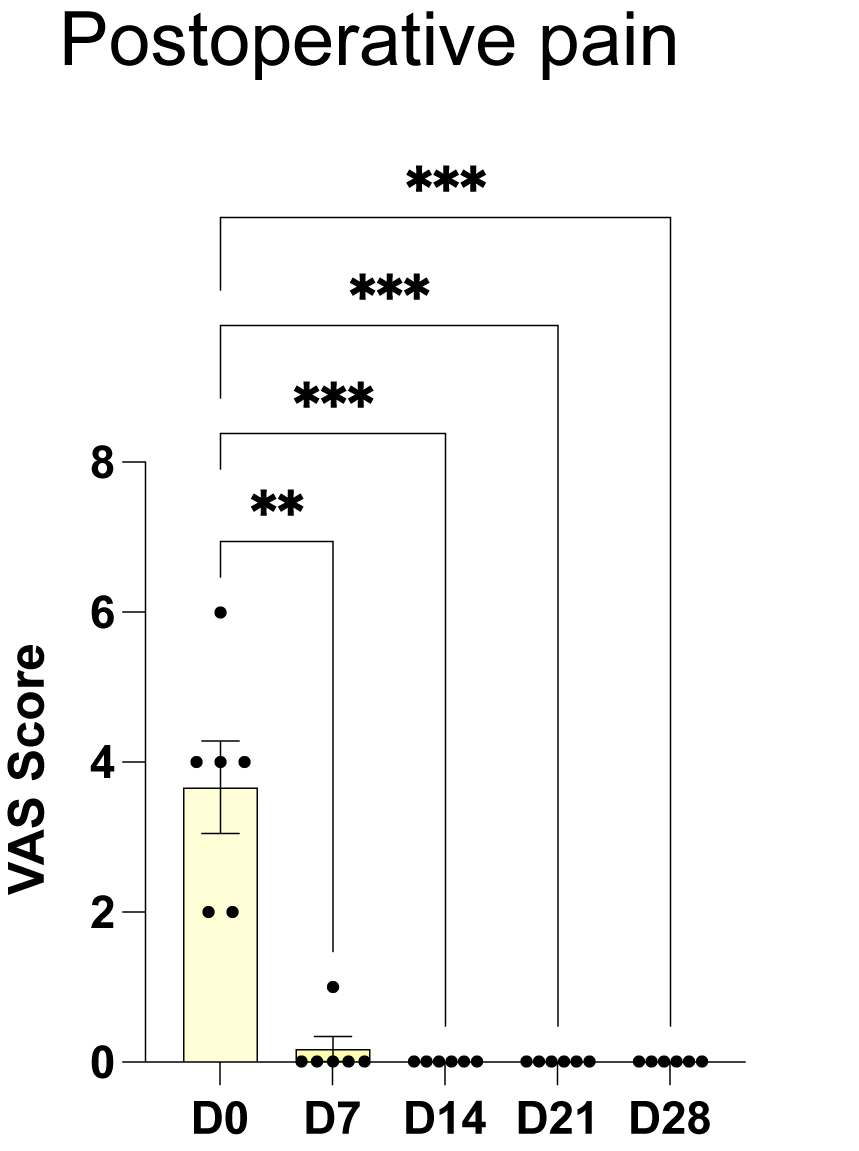

Supplement: Supplementary file 2 [file Image1.tif]
